# Structural Insight into the Working Mechanism of the FAD Synthetase from the Human Pathogen *Streptococcus pneumoniae*: A Molecular Docking Simulation Study

**DOI:** 10.3390/ijms24043121

**Published:** 2023-02-04

**Authors:** Sunghark Kwon

**Affiliations:** 1 Department of Biotechnology, Konkuk University, Chungju 27478, Chungbuk, Republic of Korea; naritsuru@kku.ac.kr; 2 Research Institute for Biomedical & Health Science, Konkuk University, Chungju 27478, Chungbuk, Republic of Korea

**Keywords:** flavin adenine dinucleotide synthetase, riboflavin phosphorylation, flavin mononucleotide adenylylation, human pathogen, *Streptococcus pneumoniae*, molecular docking

## Abstract

Flavin adenine dinucleotide synthetases (FADSs) catalyze FAD biosynthesis through two consecutive catalytic reactions, riboflavin (RF) phosphorylation and flavin mononucleotide (FMN) adenylylation. Bacterial FADSs have RF kinase (RFK) and FMN adenylyltransferase (FMNAT) domains, whereas the two domains are separated into two independent enzymes in human FADSs. Bacterial FADSs have attracted considerable attention as drug targets due to the fact that they differ from human FADSs in structure and domain combinations. In this study, we analyzed the putative FADS structure from the human pathogen *Streptococcus pneumoniae* (*Sp*FADS) determined by Kim et al., including conformational changes of key loops in the RFK domain upon substrate binding. Structural analysis and comparisons with a homologous FADS structure revealed that *Sp*FADS corresponds to a hybrid between open and closed conformations of the key loops. Surface analysis of *Sp*FADS further revealed its unique biophysical properties for substrate attraction. In addition, our molecular docking simulations predicted possible substrate-binding modes at the active sites of the RFK and FMNAT domains. Our results provide a structural basis to understand the catalytic mechanism of *Sp*FADS and develop novel *Sp*FADS inhibitors.

## 1. Introduction

Flavin adenine dinucleotide (FAD) is an essential compound as a cofactor for numerous flavoenzymes [1,2,3]. Bacterial FAD synthetases (FADSs) catalyze the biosynthesis of FAD in two consecutive reactions, which comprise riboflavin (RF) phosphorylation and flavin mononucleotide (FMN) adenylylation [4,5,6,7,8,9]. FMN is produced by a phosphate group from adenosine triphosphate (ATP) to RF, and can subsequently be converted into FAD via FMN adenylylation at the expense of another ATP. FMN adenylylation can be reversed, resulting in FMN production from FAD. In eukarya and archaea, RF phosphorylation and FMN adenylylation are performed by two enzymes, riboflavin kinase (RFK) and FMN adenylyltransferase (FMNAT), respectively [4,5,6,7,8,9]. These enzymes require a Mg ion as a cofactor for catalytic activity. Bacterial FADSs have attracted considerable attention as drug targets because of their differences from human FADSs in terms of structure and domain combinations [10].

Previous studies have elucidated FADS structures from several species [11,12,13,14]. Several FADS structures from the hyperthermophilic bacterium *Thermotoga maritima* were reported first, including its native (PDB ID: 1MRZ) [11] and compound-bound forms (PDB ID: 1S4M, 1T6X, 1T6Y, and 1T6Z) [12]. The complex forms include flavin (PDB ID: 1S4M) [12], ADP (PDB ID: 1T6X), ADP-AMP-FMN (PDB ID: 1T6Y), and RF (PDB ID: 1T6Z). FADS structures from *Corynebacterium ammoniagenes* (*Ca*FADS) have also been determined, with its native (PDB ID: 2X0K) [13], ADP-bound (PDB ID: 5A88) [14], and FMN-ADP-bound (PDB ID: 5A89 and 5A8A) forms [14]. In addition, a putative FADS structure from the human pathogen *Streptococcus pneumoniae* (*Sp*FADS; PDB ID: 3OP1) has been reported as a native form. Besides these crystallographic studies, a previous study using atomic force microscopy (AFM) produced accurate topography maps that visualized the transitional conformations of ferredoxin-dependent flavin thioredoxin reductases under the relevant conditions involved in catalysis [15]. Another study showed that the rates of solution-based reactions of the human apoptosis-inducing factor, which is a flavoreductase, were determined by using steady-state and stopped-flow spectrophotometry [16]. AFM and stopped-flow techniques were also successfully employed in a *Sp*FADS study [17]. In this paper, the authors verify how the presence of RF and FMN impacts the morphology and enzymatic reaction kinetics of *Sp*FADS, respectively [17]. 

These previous studies accompanying biochemical and biophysical analyses have unveiled the structural and functional features of FADSs [11,12,13,14]. In general, prokaryotic FADSs consist of independent N- and C-terminal domains that are responsible for FMN adenylylation and RF phosphorylation, respectively [11,12,13]. The N-terminal domain exhibits a typical Rossmann fold, whereas the C-terminal domain mainly comprises a central β-barrel with a long terminal α-helix [11,12,13]. These structural differences appear to be related to the respective catalytic functions. Considering that prokaryotic FADSs have two separate active sites, it is possible to design active site-specific inhibitors to block the two catalytic activities.

To date, studies on FADSs have been limited to those from the species mentioned above. Interestingly, the crystal structure of *Sp*FADS (PDB ID: 3OP1), determined by Kim et al., is the only known FADS structure from a human pathogen. In addition, the working mechanism of *Sp*FADS at the molecular level remains largely unknown, owing to the scarcity of information on its substrate complex structures. As a result, progress on the detailed working mechanism of *Sp*FADS has been hindered. Therefore, the elucidation of the catalytic mechanism accompanying the substrate-binding mode of *Sp*FADS is necessary for the development of inhibitor agents, as well as for a comprehensive understanding of *Sp*FADS.

Herein, we present possible substrate-binding modes of *Sp*FADS through molecular docking simulations. Structural analysis and molecular modeling propose sequential catalytic reaction mechanisms after substrate binding. In addition, our results show how RF can bind to the active site in the RFK domain for RF phosphorylation, and help to explain why the binding of FMN to the active site in the FMNAT domain occurs after ATP binding for FMN adenylylation. The present study illustrates virtual consecutive substrate-binding modes for the enzymatic activities of *Sp*FADS. The generated molecular models provide a structural basis for the development of inhibitors to specifically target *Sp*FADS.

## 2. Results and Discussion

### 2.1. Structural Features of SpFADS

**Figure 1:**
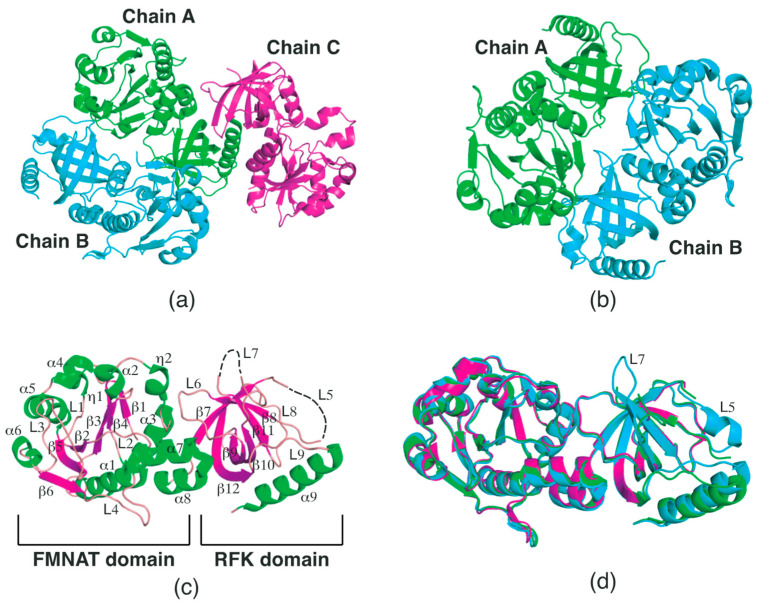
Overall structure of *Sp*FADS. (**a**) Crystal structure of *Sp*FADS in the asymmetric unit. The three monomers (Chains A–C) are represented by cartoons and colored green, cyan, and magenta, respectively. (**b**) Dimeric structure (chains A and B). (**c**) Monomeric structure (chain B). Disordered regions are depicted as black dashed lines. (**d**) Structural comparison between chains A–C. The color code is the same as in panel (**a**).

The crystal structure of *Sp*FADS was previously determined by Kim et al. (PDB ID: 3OP1). This structure, entitled ‘Crystal Structure of Macrolide-efflux protein SP_1110 from *Streptococcus pneumoniae*’, was deposited in the PDB in 2010. This structure contains three chains in the asymmetric unit (Figure 1a). The PDBePISA server [18] predicted that the most probable multimeric state of *Sp*FADS in solution is a dimer formed between chains A and B (Figure 1b). As a monomer, the overall structure of *Sp*FADS comprises N-terminal FMNAT and C-terminal RFK domains (Figure 1c). The FMNAT domain (residues 1–185) consists of ten α-helices (α1-8), two 3_10_-helices (η1-2), six β-strands (β1-6), and four loops (L1-4), adopting a typical Rossmann fold (Figure 1c). The six β-strands in the center form a twisted β-sheet, in which the β1-3 strands run parallel to each other and the β3 strands run antiparallel to the β4-6 strands. The ten α-helices surround this β-sheet, forming an α-β-α sandwich fold. The C-terminal RFK domain consists of one α-helix (α9), six β-strands (β7-12), and five loops (L5-9) (Figure 1c). The six β-strands form a β-barrel, next to which the α11 is located perpendicular to the barrel axis. Part of the L5 connecting β7 to β8, along with the L7 loop connecting β10 to β11, appears disordered in chains A and C, but not for chain B, in this crystal structure (Figure 1d), implying that these loops are intrinsically flexible regions. Previous studies have revealed that the L5 and L7 loops in the RFK domain are associated with the formation of the active site, which consists of riboflavin-binding (R-pocket) and ATP-binding (A-pocket) pockets [11,12,13,14].

**Figure 2:**
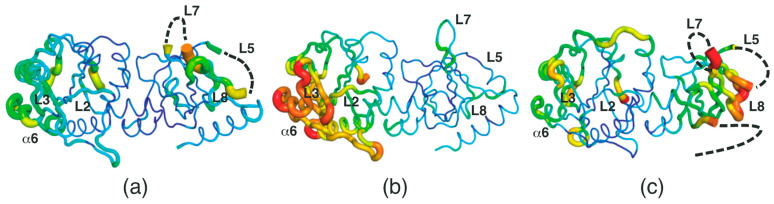
*B*-factor distribution of *Sp*FADS. The monomeric structure in chains A-C (**a**–**c**) is shown by a putty representation and rainbow-colored from red to violet in order of *B*-factor value. The dashed lines indicate disordered regions.

Considering that *Sp*FADS has several loops in the respective domains, its flexibility needs to be investigated, as these loops may play vital roles in the catalytic reaction. Thus, we analyzed the *B*-factor distribution of the *Sp*FADS structure. This analysis showed that the *Sp*FADS structure exhibits somewhat a different *B*-factor distribution, depending on each molecule in the asymmetric unit. Compared with chain A (Figure 2a), the α6, L2, and L3 regions in chain B exhibited high *B*-factor values (Figure 2b), whereas loop regions, such as L5, L7, and L8 in chain C, showed high values (Figure 2c). Notably, the L5 and L7 regions were disordered, suggesting that these regions retained their intrinsic flexibility (Figure 2c). These results can likely be attributed to the position of the three molecules in different crystallographic environments.

Structural comparison of the three chains further confirmed that the L5 and L7 loops in the RFK domain adopt different conformations depending on the crystallographic environment. Specifically, the L5 loop can adopt a short helix form in a structurally stable condition, while the L7 loop constitutes a β-hairpin along with the two extended β-strands (Figure 1d). These diverse conformations are assumed to play key roles in the construction of the active site.

### 2.2. Conformational Changes in the RFK Domain

**Figure 3:**
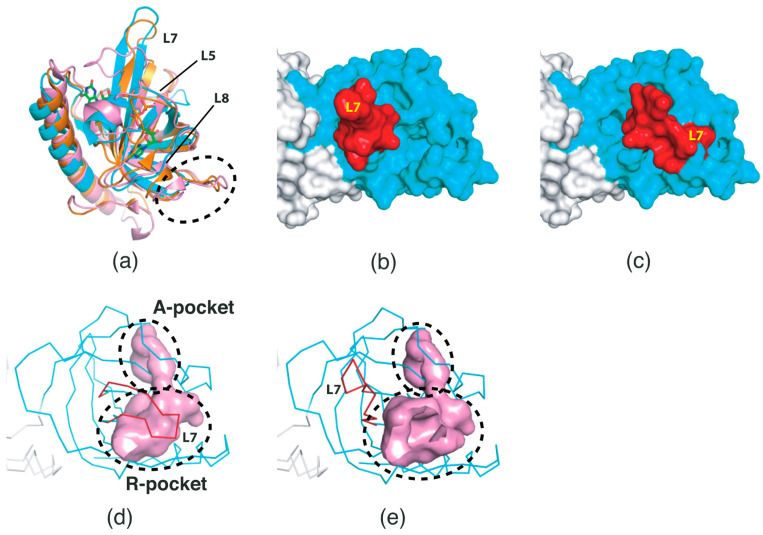
Conformational changes of *Sp*FADS. (**a**) Structural comparison between *Sp*FADS (cyan) and *Ca*FADS in the native (brown; PDB ID: 2X0K) and FMN-ADP-bound (pink; PDB ID: 5A8A) forms. The two structures of *Ca*FADS are superimposed onto that of *Sp*FADS (chain B). The FMN and ADP molecules are shown in stick representation and colored green. The black dashed ellipse indicates the β-hairpin region connecting β9 and β10 of *Sp*FADS. The RFK domain (cyan) of *Sp*FADS is shown in its open (**b**) and closed (**c**) forms. The L7 loop regions, including β10 to β11, are colored red. Two pockets in the RFK domain are shown in their open (**d**) and closed (**e**) forms. The black dashed ellipses indicate the two pockets. Their cavities are colored pink.

Previous studies on *Ca*FADS structures have provided valuable information on the conformational changes in loops positioned in the proximity of the active site in the RFK domain [13,14]. These studies showed that two regions, corresponding to the L5 and L7 loops of the *Sp*FADS structure (chain A), adopt different conformations [13,14]. The native form of *Ca*FADS (PDB ID: 2X0K) exhibits an open conformation in the two loop regions [13], whereas its FMN-ADP-bound form (PDB ID: 5A8A) adopts a closed conformation (Figure 3a) [14]. These conformational changes indicate that *Ca*FADS, maintaining an open conformation in the “resting state”, binds its substrates in the active site, thereafter adopting a closed conformation. Therefore, this FMN-ADP-bound structure (PDB ID: 5A8A) [14] constitutes a snapshot of the state prior to the final step of FMN product release.

We compared the RFK domain structure of *Sp*FADS (chain B) with that of *Ca*FADS (PDB ID: 2X0K and 5A8A) [13,14]. Although an overall similar architecture is evident, *Sp*FADS is structurally different in the L5, L7, and L8 loop regions (Figure 3a). Notably, *Sp*FADS showed a hybrid structure between the open and closed conformations of *Ca*FADS (Figure 3a). While the location of the L5 loop of *Sp*FADS is nearly the same as that of the FMN-ADP-bound form of *Ca*FADS (PDB ID: 5A8A) [14], the L7 loop adopts an open conformation, similar to that of the native form of *Ca*FADS (PDB ID: 2X0K) (Figure 3a) [13]. Such conformational diversity implies that these regions have intrinsically substantial flexibility, and that the optimal conformation may be selected in response to the substrates. In addition, we found that the β-hairpin region connecting β9 and β10 in the *Sp*FADS structure corresponds to a relatively long loop in the *Ca*FADS structure (Figure 3a), likely due to genetic differences in the corresponding region between the two species.

To assess the architecture of the active site, we rebuilt the L5 loop of *Sp*FADS in its closed form based on the atomic coordinates of *Ca*FADS. Surface representation distinctly showed that the entrance to the active site of *Sp*FADS is structurally different between the open and closed forms. In the open form, the entrance to the active site was exposed to the outer environment (Figure 3b), whereas it was shielded by the L7 loop in the closed form (Figure 3c). Therefore, the L7 loop probably plays a vital role in the formation of the active site and occlusion of outer molecules during catalysis.

It is reasonable to assume that the conformational change of the L7 loop is reflected in volumetric differences in the active site. Indeed, volumetric analysis revealed that the volume of the active site in the closed form is smaller than that in the open form (Figure 3d,e). Specifically, the volume of the R-pocket appeared to be decreased, whereas that of the A-pocket was nearly constant (Figure 3d,e). This result implies that potential inhibitors targeting *Sp*FADS should be precisely designed based on the architecture of the active site in the closed form.

### 2.3. Surface Properties of SpFADS

**Figure 4:**
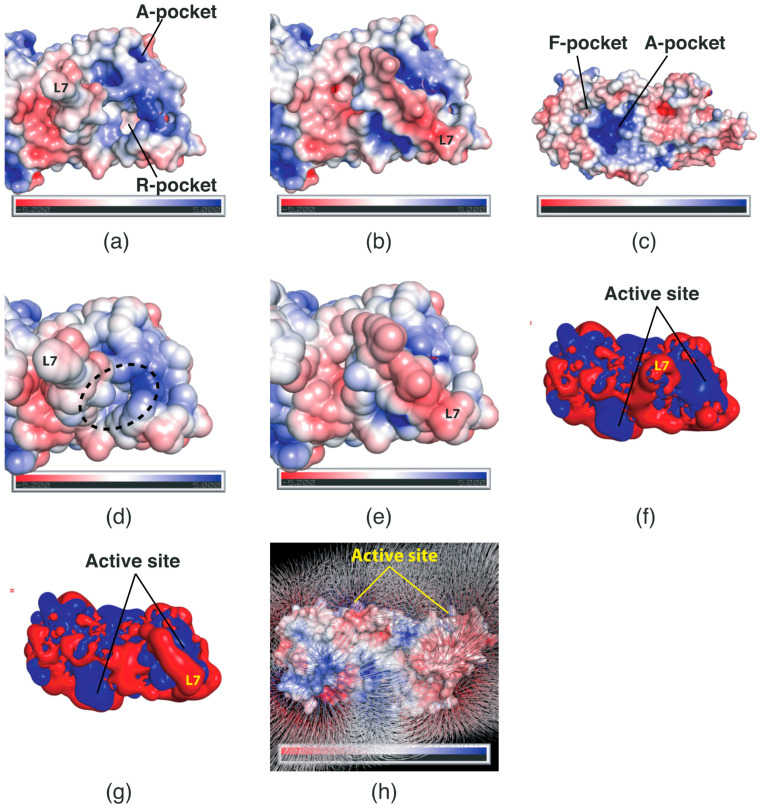
Surface electrostatic potential of *Sp*FADS. Electrostatic potential of the RFK domain in the open (**a**) and closed (**b**) forms, as well as the FMNAT domain (**c**). The scale bar ranges from −5 kT/e (red) to 5 kT/e (blue). Solvent-accessible surface area of the RFK domain in the open (**d**) and closed (**e**) forms. Electrostatic potential isocontour of the RFK domain in the open (**f**) and closed (**g**) forms. The isocontour map ranges from −1 kT/e (red) to +1 kT/e (blue). (**h**) Electric field generated by the surface electrostatic potential of *Sp*FADS. Surface electrostatic potential is the same as in panels (**a**–**c**). The electric field map is contoured and depicted at the −0.5 σ level.

To obtain biophysical insights into the substrate attraction of *Sp*FADS, the surface charge distribution of *Sp*FADS was investigated. We analyzed the surface electrostatic potential based on the numerical solution of the Poisson–Boltzmann equation [19]. Our analysis showed that relatively neutral residues are distributed in the R-pocket in the open form of the RFK domain, while a distribution of positively charged residues dominates the A-pocket (Figure 4a). These charge distributions seem reasonable, considering that riboflavin is electrostatically neutral, whereas the ionized ATP molecule has three negative charges. In the closed form of the RFK domain, the entrance to the active site was blocked by the L7 loop (Figure 4b). The rear of the L7 loop exhibits a negatively charged area, which constitutes a portion that is exposed to the outer water-soluble environment (Figure 4b). In addition, the A-pocket in the FMNAT domain distinctly showed a positively charged area, whereas the F-pocket displayed relatively neutral surface potential (Figure 4c). This potential distribution probably facilitates the binding of ATP to the A-pocket.

Although an open entrance to the active site was observed, it is necessary to determine whether substrates are accessible to the two pockets at the active site in the RFK domain. Hence, we assessed solvent-accessible surfaces in the proximity of the active site. Our analysis clearly revealed that the entrance to the active site in the open form is spatially adequate to accept the substrates (Figure 4d). A sufficient gap was noted between the solvent-accessible surfaces of the L5 and L7 loops. This space is assumed to facilitate the substrate access to the active site. On the other hand, in the closed form, the entrance to the active site was thoroughly closed by the L7 loop (Figure 4e), thereby disrupting the access of the substrate, including water molecules, to the active site.

Electrostatic potential isocontour analysis provides additional insight into substrate attraction to the active site. An electrostatic potential isocontour map of *Sp*FADS revealed the electrostatic surface properties. Specifically, a positively charged region was formed on the surface of the active site in the RFK domain, whereas the L7 loop region exhibited a negative potential (Figure 4f). These unique charge distributions imply that the positively charged active site can attract negatively charged substrates, such as ATP, and that subsequently, the L7 loop covers the active site through electrostatic attraction. Interestingly, we found that the rear of the active site exhibited a wide negative potential isocontour map (Figure 4g). This antithetical distribution suggests that the RFK domain may exploit the electric field generated by the two regions to attract its substrate to the active site. Indeed, our electric field simulation showed that the rear area can generate a strong electric field to attract negatively charged molecules (Figure 4h).

### 2.4. Oxidized and Reduced RF-Binding Modes in the RFK Domain

**Figure 5:**
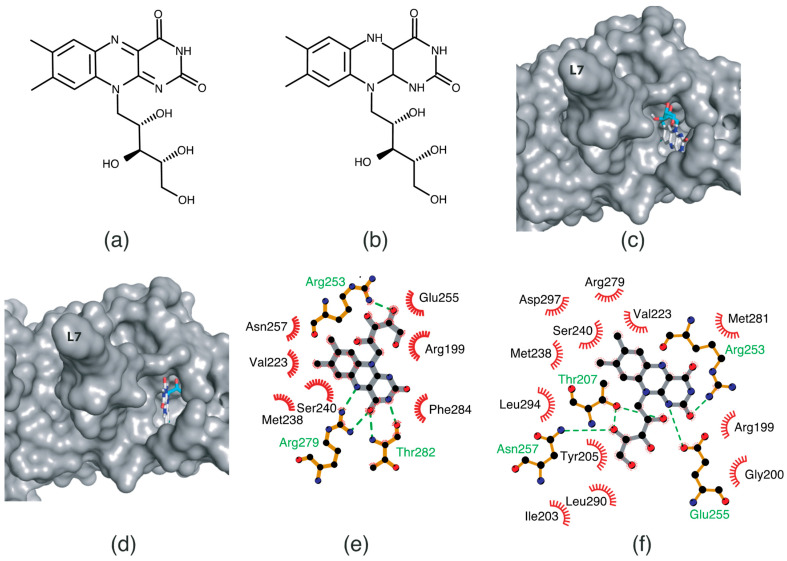
Molecular docking analysis of RF in the oxidized and reduced forms. The RF molecular structures in the oxidized (**a**) and reduced (**b**) forms. Docking results of RF in the oxidized (**c**) and reduced (**d**) forms. *Sp*FADS and RF are shown by cartoon and stick representations, respectively. The isoalloxazine ring and ribityl chain of RF are colored white and blue, respectively. Schematic diagrams of RF in the oxidized (**e**) and reduced (**f**) forms and its interactions with adjacent residues. The green dashed lines indicate hydrogen bonds.

The RF molecule can be interconverted between four redox forms: flavine-N(5)-oxide (superoxidized form), quinone (oxidized form; Figure 5a), semiquinone (half-reduced form), and hydroquinone (reduced form; FADH_2_; Figure 5b) by accepting and donating protons and electrons [20,21,22,23]. Quinone is the most thermodynamically stable form, as it constitutes an aromatic compound with resonance structures. FADH_2_ is relatively unstable owing to its low aromaticity. However, it needs to be structurally explained which form *Sp*FADS prefers as its substrate during the first catalytic reaction for FMN production. Thus, we conducted molecular docking simulations with the quinone and hydroquinone forms of RF, targeting the active site in the RFK domain of *Sp*FADS.

The RF structure was prepared with oxidized (Figure 5a) and reduced (Figure 5b) forms for the docking simulations. To generate the reduced form, hydrogen atoms were added to two nitrogen atoms (N1 and N5). Several residues in the active site (Ile203, Tyr205, Thr207, Asn209, Val223, Ser240, Glu255, Arg279, Thr282, Phe284, Leu290, Leu294, and Asp297) were designated as flexible residues to investigate optimal conformers in response to substrates. The RFK domain, as a molecular docking target, contains an ATP molecule in the A-pocket.

The results of the molecular docking simulation are presented in Table 1. The resulting conformers were ranked in order of low energy values. The top-ranked conformers in the oxidized and reduced forms were selected for structural analysis. The results showed that the two conformers differed in terms of binding modes. While the isoalloxazine ring moiety of RF in the oxidized form was oriented toward the inside of the binding site (Figure 5c), it was exposed to the outer entrance of the binding site in the reduced form (Figure 5d). Moreover, the ribityl chain in the oxidized form was positioned near the γ-phosphate group of ATP (Figure 5c), but was oriented toward a deeper site in the reduced form (Figure 5d). The latter orientation likely renders the access of the ribityl chain to the γ-phosphate group of ATP difficult, thereby impeding phosphorylation.

Different binding modes are clearly illustrated in the schematic diagrams of the RF–residue interactions. In the oxidized form, the isoalloxazine ring was appropriately assigned to the R-pocket (Figure 5e). Two nitrogen atoms and one oxygen atom in the isoalloxazine ring formed hydrogen bonds with Arg279 and Thr282. The oxygen atom linked to C4’ in the ribityl chain formed a hydrogen bond with Arg253. In addition, many carbon atoms in RF interacted with hydrophobic moieties of adjacent residues, such as Arg199, Val223, Met238, Ser240, Glu255, Asn257, and Phe284.

In contrast, the interactions of the reduced form with adjacent residues were different (Figure 5f). Oxygen and nitrogen atoms in the isoalloxazine ring formed hydrogen bonds with Arg253 and Glu255, respectively. Two oxygen atoms in the ribityl chain formed hydrogen bonds with Thr207 and Asn257. Compared with the oxidized form, more adjacent residues were involved in hydrophobic interactions with RF in the reduced form (Figure 5f). These residues included Arg199, Gly200, Ile203, Tyr205, Val223, Met238, Ser240, Arg279, Met281, Leu290, Leu294, and Asp297. Consequently, our molecular docking analyses showed that RF in the oxidized, rather than in the reduced, form adopted a conformation suitable for phosphorylation.

### 2.5. FMN-Binding Mode in the FMNAT Domain

The crystal structure of *T. maritima* FADS in complex with AMP in the FMNAT domain has previously been reported [11,12]. However, the structure of the FMNAT domain containing two substrates (FMN and ATP) or two products (FAD and pyrophosphate [PP_i_]) has not been elucidated yet. Structural analysis of the two substrates of the FMNAT domain is necessary to better understand the catalytic mechanism of FMN adenylylation. In addition, a previous study proposed a possible kinetic mechanism for *C. ammoniagenes* FADS [4]; the authors suggested that ATP binds to the active site in the FMNAT domain before FMN [4]. Considering that the FMNAT domain has F- and A-pockets at the active site, the binding order of the two distinct substrates may be an important determinant of FMN adenylylation kinetics. This issue also needs to be addressed via structural analysis. Thus, we performed molecular docking simulations of FMN to understand how FMN and ATP bind to the respective pockets, and why FMN’s binding to the active site probably follows that of ATP.

The structure of the FMNAT domain containing ATP at the active site has not yet been determined. Thus, we built an ATP/Mg^2+^ molecular model at the A-pocket, based on the coordinates of AMP in the crystal structure of *T. maritima* FADS (PDB ID: 1T6Y). Molecular docking simulations of FMN were performed with ATP/Mg^2+^ at the A-pocket to investigate the FMN-binding mode for FMN adenylylation, and without ATP/Mg^2+^ to identify the structural basis for the order of substrate binding. In these molecular docking simulations, the grid box included both the F- and A-pockets.

**Figure 6:**
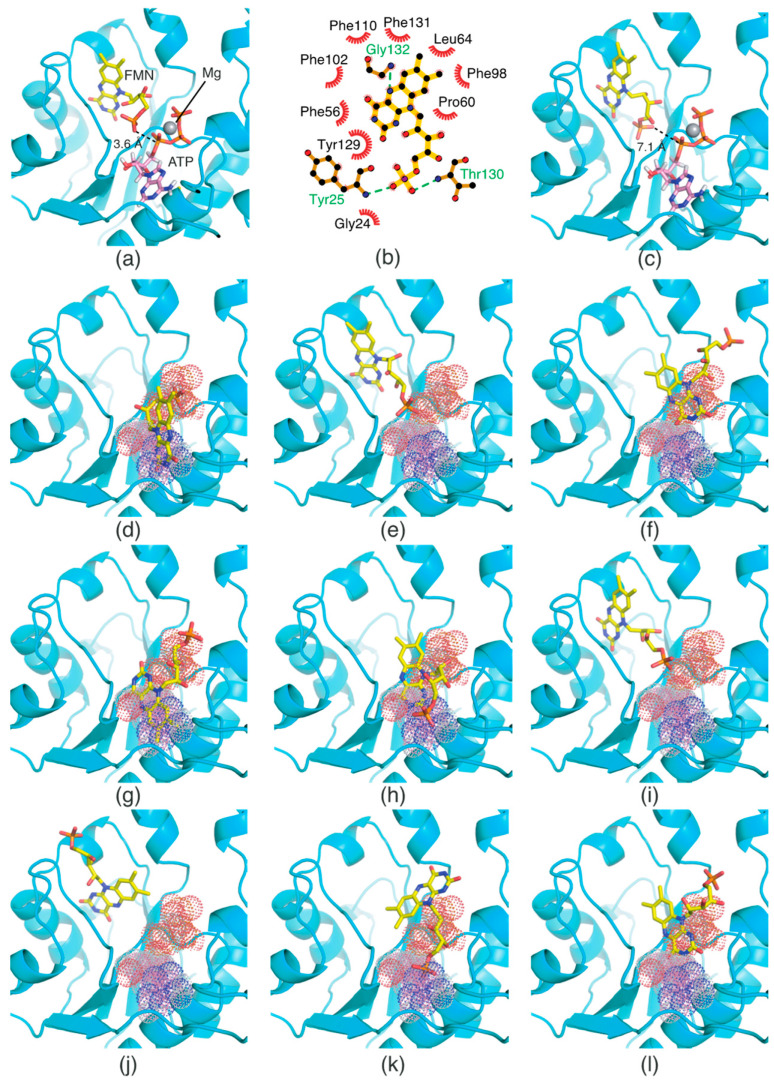
Molecular docking analysis of FMN in the presence (**a**) and absence (**c**–**l**) of virtual ATP. (**b**) A schematic diagram of FMN interactions in panel a. The ATP molecule is represented by dots in panels (**d**–**l**). The color code of the dots shown in panels d-l is the same as in panel (**c**).

The results of the docking simulations performed in the presence of ATP/Mg^2+^ are presented in Table 2. Of the resulting conformers, the top-ranked conformer was selected for structural analysis. We found that FMN bound suitably to the F-pocket (Figure 6a). Specifically, the two carbonyl groups in the isoalloxazine ring were oriented toward the inside of the pocket, whereas the phosphate group was positioned in proximity to the α-phosphate group of ATP in the A-pocket (Figure 6a). The distance between an oxygen atom in the phosphate group of FMN and the α-phosphorous atom of ATP is 3.6 Å, which seems appropriate for forming a chemical bond.

A schematic diagram of FMN binding provides detailed information on its interactions with adjacent residues (Figure 6b). The N(5) atom in the isoalloxazine ring forms a hydrogen bond with the Gly132 backbone. Two oxygen atoms in the phosphate group also form hydrogen bonds with the backbone moieties of Tyr25 and Thr130. Remarkably, FMN is surrounded by adjacent hydrophobic residues such as Gly24, Phe56, Pro60, Leu64, Phe98, Phe102, Phe110, Tyr129, and Phe131. This binding mode signifies that hydrophobic interactions are an important factor for FMN binding, similarly to RF binding in the RFK domain.

The results of the molecular docking simulation in the absence of ATP/Mg^2+^ are summarized in Table 2. Compared with the binding energy values of the FMN conformers in the presence of ATP/Mg^2+^, binding energy values were relatively high in the docking simulation performed without ATP/Mg^2+^. This result indicates that FMN thermodynamically favors a binding condition with ATP/Mg^2+^ to one without ATP/Mg^2+^. The docking results also showed that FMN without ATP/Mg^2+^ adopts inappropriate conformations for catalysis in terms of both orientation and position (Figure 6c–l). Specifically, the phosphate group of the top-ranked conformer (C1) was oriented toward the bottom of the F-pocket (Figure 6c), compared with that of the conformer in the presence of ATP/Mg^2+^ (Figure 6a). The distance between an oxygen atom in the phosphate group and the α-phosphorous atom of virtual ATP is 7.1 Å, which appears to somewhat exceed the optimal distance for catalysis. Thus, this orientation renders the catalytic reaction challenging. Other conformers (C2, C4, C5, C6, C9, and C10) bound to the A-pocket, but not the F-pocket (Figure 6d,f–h,k,l). These results suggest that FMN may compete with ATP for binding, thereby obstructing the binding of ATP to the A-pocket. In the C3 and C7 conformers, steric clashes were observed between the phosphate group and virtual ATP (Figure 6e,i). Lastly, the phosphate group of the C8 conformer was oriented toward the outside of the F-pocket (Figure 6j). Consequently, in the absence of ATP/Mg^2+^, none of the conformers bound to the F-pocket suitably for FMN adenylylation. These docking simulation results explain why ATP binding prior to FMN is a prerequisite for FMN adenylylation.

### 2.6. Pharmacophore Model

To date, commercially available inhibitors for *Sp*FADS have not been developed. However, a previous study showed that several compounds obtained using high-throughput screening (HTS) have an antimicrobial effect on both *Ca*FADS and *Sp*FADS, which means that they may lead to the development of antibacterial drugs targeting *Sp*FADS [24]. These compounds inhibited the RFK and FMNAT activities. However, most compounds targeted the FMNAT activity. Hence, nine compounds inhibiting the FMNAT activity of *Sp*FADS [24] were selected to build the pharmacophore model. The compound structures are presented in Table 3.

**Figure 7:**
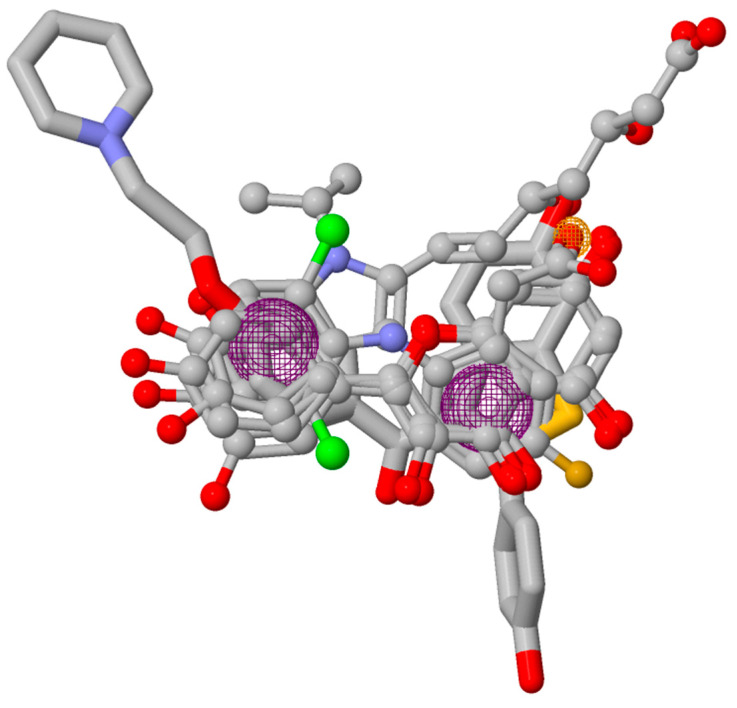
The pharmacophore model for *Sp*FADS. Six compounds were superimposed. Pharmacophore features such as aromatic rings (magenta) and a hydrogen bond acceptor (yellow) are depicted.

A total of eleven pharmacophore models were generated from nine input compounds. The top-scoring model exhibiting the highest score value (23.062) was selected as the best pharmacophore model, which included pharmacophoric features such as two aromatic rings and one hydrogen bond acceptor (Figure 7). However, this pharmacophore model was built based on HTS hit compounds targeting *Ca*FADS, not *Sp*FADS. Thus, it is possible to detect other pharmacophoric features if HTS is conducted targeting *Sp*FADS. Nevertheless, this result may provide valuable information on spatial features which *Sp*FADS inhibitors should include.

## 3. Materials and Methods

### 3.1. Protein and Substrate Preparation

Structure files for *Sp*FADS and its substrates were retrieved from the Protein Data Bank (https://www.rcsb.org (accessed on 26 November 2022)). Water molecules involved in the protein structure were removed, and hydrogen atoms were added to the protein for molecular docking simulations.

### 3.2. Molecular Docking Simulation

To calculate the charge of the *Sp*FADS structure, the Gasteiger charges [25] were applied. PDBQT files for *Sp*FADS and its substrates were generated using AutoDockTools 1.5.6 [26]. Residues comprising the active sites of *Sp*FADS were designated as flexible residues to increase the accuracy of molecular docking simulations. The flexible residues were embedded in a virtual grid box for docking calculation. The grid size along the x, y, and z dimensions was set to 20 Å × 20 Å × 20 Å. The grid box spacing was adjusted to 1 Å. Other parameters for molecular docking calculation were set to default values. Molecular docking simulation was performed using AutoDock Vina [27,28]. Ten different conformers per docking simulation were generated in order of low binding energy scores. The top-ranked conformers were selected as docking models.

### 3.3. 3D-Pharmacophore Modeling

PharmaGist, a program for pharmacophore detection [29], was used to build the pharmacophore model for *Sp*FADS inhibitors. The program determines pharmacophobic features using a ligand-based method and computes using multiple alignment of flexible ligands. The alignment process is performed with pivot iteration of pairwise and multiple alignments. Nine *Ca*FADS inhibitors were selected as input molecules for the pharmacophore model construction. The best pharmacophore model of the candidate pharmacophores was determined based on the score.

### 3.4. Structure Visualization

All structural figures shown in this paper were generated using PyMOL [30] and LigPlot+ [31].

## 4. Conclusions and Future Perspectives

In the current study, we found that the key loops in the RFK domain probably play a pivotal role in RF phosphorylation by adopting open and closed conformations. This finding provides structural insight into the design of novel inhibitors to target *Sp*FADS. Namely, the structure of the active site in the closed, rather than in the open, form appears to be suitable for substrate conformation in the transition state. In addition, surface potential analysis suggests that electrostatic interactions may be key factors for substrate attraction. Our molecular docking simulations provide a structural basis to understand the two sequential catalytic reactions, RF phosphorylation and FMN adenylylation, and why *Sp*FADS prefers FMN in the reduced form as its substrate.

*Sp*FADS recognizes RF and ATP in the RFK domain, and FMN and ATP in the FMNAT domain, as its substrates for catalysis. To comprehend these two catalytic reactions at the molecular level, various analytical methods are required. Given that crystallization of *Sp*FADS in a complex with its heterogeneous substrates is difficult to achieve, computational tools such as molecular docking simulation could be alternatives to illustrate its substrate-binding modes. In this context, our molecular docking simulation results provide structural insight into a plausible working mechanism of *Sp*FADS, concerning its substrate recognition. However, our docking simulation was performed in a vacuum state, and the protein was considered a rigid body. Therefore, further studies should focus on analysis of dynamic properties of *Sp*FADS in a solvated state using molecular dynamics simulation, including the molecular mechanics Poisson–Boltzmann Surface Area approach.

Antibiotic resistance has become a severe threat to global health, and urges the development of novel antibiotics. Considering that antibiotic-resistant pathogenic bacteria are dependent on FADSs to maintain metabolic homeostasis, FADSs can constitute attractive drug targets against antibiotic-resistant pathogens. In addition, FADSs have attracted attention in that they serve as bioreactors to catalyze the conversion of RF to FMN [32]. A deeper understanding of the working mechanism of FADSs will lead to a wide range of applications of FADSs in the development of novel antimicrobial agents and biomaterial synthesis.

## Figures and Tables

**Table 1:** Molecular docking simulation results of RF in the oxidized and reduced forms.

Rank	Binding Energy (kcal/mol) (Oxidized Form)	Binding Energy (kcal/mol) (Reduced Form)
1	−9.0	−9.4
2	−9.0	−9.2
3	−9.0	−9.1
4	−8.9	−8.9
5	−8.8	−8.8
6	−8.6	−8.7
7	−8.6	−8.2
8	−7.8	−7.3
9	−7.4	−7.3
10	−7.1	−7.2

**Table 2:** Molecular docking simulation results of FMN in the presence and absence of ATP.

Rank	Conformer	Binding Energy (kcal/mol) (with ATP)	Binding Energy (kcal/mol) (without ATP)
1	C1	−9.1	−8.9
2	C2	−9.1	−8.8
3	C3	−9.0	−8.8
4	C4	−8.8	−8.7
5	C5	−8.8	−8.6
6	C6	−8.7	−8.5
7	C7	−8.6	−8.1
8	C8	−8.4	−8.1
9	C9	−8.3	−8.1
10	C10	−8.1	−8.1

**Table 3:** Compounds selected to build the pharmacophore model for *Sp*FADS inhibitors.

Compound	Chemical Structure
1	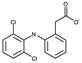
2	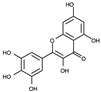
3	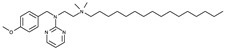
4	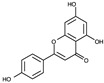
5	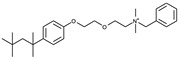
6	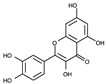
7	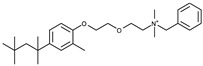
8	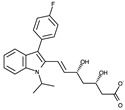
9	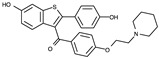

## Data Availability

Not applicable.
